# Viewpoint Analysis for Maturity Classification of Sweet Peppers

**DOI:** 10.3390/s20133783

**Published:** 2020-07-06

**Authors:** Ben Harel, Rick van Essen, Yisrael Parmet, Yael Edan

**Affiliations:** 1 Dept. of Industrial Engineering and Management, Ben-Gurion University of the Negev, Beer Sheva 8410501, Israel; rick.vanessen@wur.nl (R.v.E.); iparmet@gmail.com (Y.P.); yael@bgu.ac.il (Y.E.); 2 Farm Technology Group, Wageningen University and Research, 6700 AA Wageningen, The Netherlands

**Keywords:** viewpoint analysis, camera position, maturity classification, machine vision, sweet pepper

## Abstract

The effect of camera viewpoint and fruit orientation on the performance of a sweet pepper maturity level classification algorithm was evaluated. Image datasets of sweet peppers harvested from a commercial greenhouse were collected using two different methods, resulting in 789 RGB—Red Green Blue (images acquired in a photocell) and 417 RGB-D—Red Green Blue-Depth (images acquired by a robotic arm in the laboratory), which are published as part of this paper. Maturity level classification was performed using a random forest algorithm. Classifications of maturity level from different camera viewpoints, using a combination of viewpoints, and different fruit orientations on the plant were evaluated and compared to manual classification. Results revealed that: (1) the bottom viewpoint is the best single viewpoint for maturity level classification accuracy; (2) information from two viewpoints increases the classification by 25 and 15 percent compared to a single viewpoint for red and yellow peppers, respectively, and (3) classification performance is highly dependent on the fruit’s orientation on the plant.

## 1. Introduction

Robotic harvesting can help overcome the lack of manual and seasonal labor, reduce production costs, and increase the quality of the harvested product [1]. For successful robotic harvesting, the robot must detect the fruit, reach the fruit, determine if the fruit is mature, detach the mature fruit from the plant, and transfer it to a container [2]. Most agricultural robotics research and development projects [3,4,5] focused on detecting [6,7,8], reaching [4,9,10], and detaching the fruit [4,9], with only a few studies focusing on maturity level determination [11,12,13]. Since different fruits can be in different maturity stages within the field and even on the same plant/branch, maturity classification is essential to enable selective harvesting [3] and an important element of an intelligent fruit-picking robot. 

In precision agriculture, maturity classification is important in phenotyping [14], and mapping yield and quality for management decisions (e.g., irrigation, fertilization [15]). Maturity is an important factor in determining the storage life and ripening quality of fruits [16] and influences the market value and choice of the consumer [17]. Several characteristics determine fruit maturity status, such as color, aroma, size, firmness, sugar content, and acidity [18,19]. Sweet peppers (*Capsicum annuum* L.) are widely cultivated since they are rich in flavor and a good source of vitamin C, which is known for its antioxidant activity [20,21]. Human harvesters usually estimate the maturity level of sweet peppers via the percentage of the pepper that has changed color from green to red/yellow [22]. The coloring percentage of pepper is highly correlated to other attributes of maturity, such as sugar content and firmness [22]. However, sweet peppers do not ripen uniformly [21,23]. Since the whole pepper must be examined in order to estimate its color percentage, the precise determination of sweet pepper fruit maturity level is difficult before harvest [22], because when the pepper is still attached to the plant, not the whole surface is visible.

**Figure 1:**
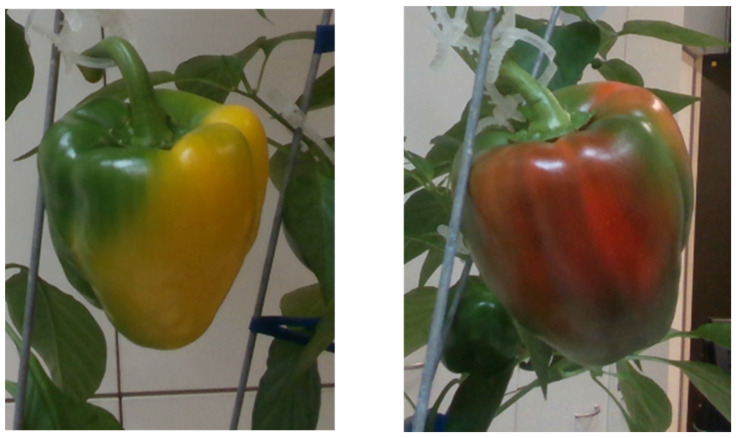
Example of the nonuniform change of color during the red and yellow pepper ripening process.

Image processing and machine vision for the maturity level classification of fruits have been intensively investigated [24,25,26,27,28,29,30,31]. Most work to date has focused on maturity analysis of fruit that ripen in a uniform fashion, such as tomato [32,33,34], passion fruit [27], apricot [24], persimmon [35], blueberry [36,37], cherry [38], and date [39]. Different methods were used for classification (e.g., support vector machines [27,36], convolutional neural networks [34,39], random forest [40], K-nearest neighbor [33], and linear discriminant analysis [35]) based on different sensors (e.g., RGB—Red Green Blue [29,33,35,36], RGB-D—Red Green Blue-Depth [27], and NIR—Near Infra-Red [38]). The current research used a RGB camera and focused on the maturity level classification of sweet peppers, which have a nonuniform ripening pattern [21,23] Figure 1). Furthermore, most of the papers above focused on image processing aspects of maturity level determination as the primary goal. The focus of the current research was to evaluate the effects of camera viewpoint and fruit orientation on the performance of a sweet pepper maturity level classification algorithm. 

In the fruit grading process, multiple viewpoints or multiple cameras are used for maturity assessment [41]. However, equipping a harvester robot with multiple cameras can be expensive, and acquiring multiple viewpoints before harvesting a single fruit could be time consuming, leading to increased cycle times [6]. Therefore, research on the best viewpoints to estimate fruit maturity while using the minimal number of viewpoints is essential for the development and the cost effectiveness of harvesting robots.

Furthermore, due to the occlusion of sweet peppers by leaves and other plant parts, only some of the fruits are visible from one viewpoint [42,43,44]. By combining different viewpoints, the number of detected peppers can be improved from 40–60% up to 85% [42,43,44,45]. In addition to their number, the choice of viewpoints also influences the detectability of the peppers [43]. Detectability varies significantly between different viewpoints, with up to 50% differences [43]. Therefore, choosing the best viewpoint and the best number of viewpoints is essential for detection [6,43].

The objective of this research is to examine the effect of camera viewpoint and fruit orientation on the performance of sweet pepper maturity level classification. The specific objectives are to:

1. Determine if there is a significant difference between sweet pepper viewpoints in providing maturity-related information, and in maturity level classification;
2. Analyze multiple viewpoint combinations for maturity level classification;
3. Study the dependence of maturity level classification on fruit orientation on the plant.

## 2. Materials and Methods

The viewpoint analyses included three parts, all of which were conducted in laboratory conditions with sweet peppers acquired from a commercial greenhouse. First, the viewpoint analysis of sweet pepper images acquired in a photocell was conducted. Then, at a different time, an online experiment with a robotic arm in the laboratory was performed with peppers reattached to a pepper plant to classify the maturity level of peppers from different viewpoints. Finally, the dependence of maturity level classification on the fruit orientation on the plant was analyzed. All parts of this research used the same algorithm to fit the maturity classification models, which was developed based on previous research [46] and based on information from an RGB camera. Since the main focus was the viewpoint analyses, laboratory conditions were essential to enable a controlled experiment.

### 2.1. Data

#### 2.1.1. Ground Truth 

##### Whole Pepper Classification 

**Figure 2:**
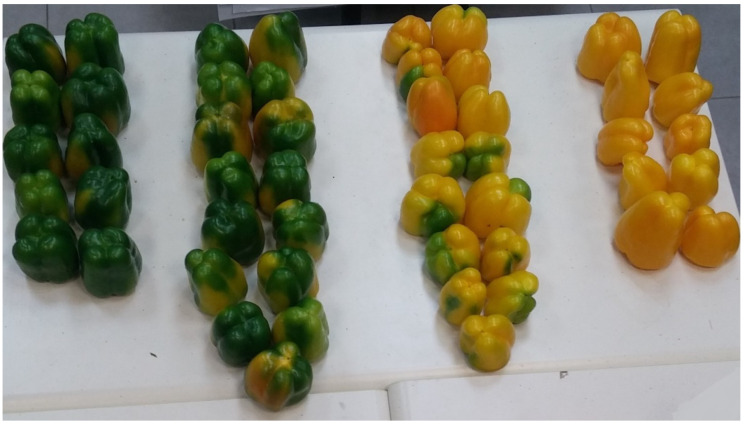
Example of peppers from the different maturity classes 1 (left) to 4 (right).

The ground truth class of each pepper in this research was determined manually as follows. The mature peppers, classes 3 and 4, were taken from the packaging house after they were harvested the same morning by professional pickers. Class 4 was determined as peppers that are entirely red/yellow colored and class 3 as peppers that included a mixture of red/yellow and green. The immature peppers, classes 1 and 2, were harvested off the plants in the same greenhouse on the same day after the pickers concluded their harvesting; this ensured the fruits are immature. Class 1 was determined as peppers that were fully green colored, and class 2 as peppers that included a mixture of green and red/yellow (Figure 2). To determine the pepper’s class, the human examined all sides of each pepper (Table 1).

##### Viewpoint Classification 

**Figure 3:**
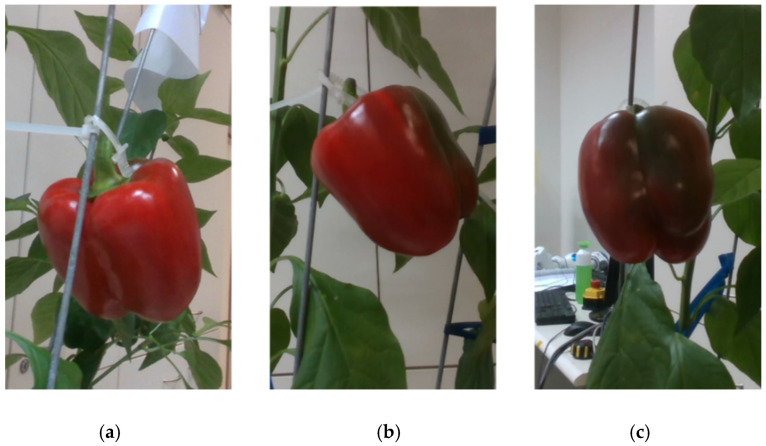
Images of a class 3 red pepper from three viewpoints in the robotic experiments: viewpoint (**a**) will be classified into class 4, (**b**) class 3, and (**c**) class 2.

Since the apparent maturity class of pepper from one viewpoint can be different from the pepper’s actual maturity class, each image was also manually classified into the maturity class that corresponds to the pepper’s appearance from that specific viewpoint (Figure 3). The viewpoint classification was conducted without knowing the whole pepper classification and was based on the following rules:

1. Class 1 if the pepper from the current viewpoint is more than 95% green.
2. Class 2 if the pepper from the current viewpoint is more than 50% green.
3. Class 3 if the pepper from the current viewpoint is more than 50% red/yellow-colored.
4. Class 4 if the pepper from the current viewpoint is more than 95% red/yellow-colored.

This viewpoint classification provides a baseline to analyze the correlation between the results from the different viewpoints and the pepper’s maturity level, as appears in the specific image.

#### 2.1.2. Data Collection

Three different datasets were collected. Each dataset was collected and measured on the same day in a few hours. Hence, there was no change in the maturity level of the peppers during the acquisition.

##### “Photocell” Dataset

**Figure 4:**
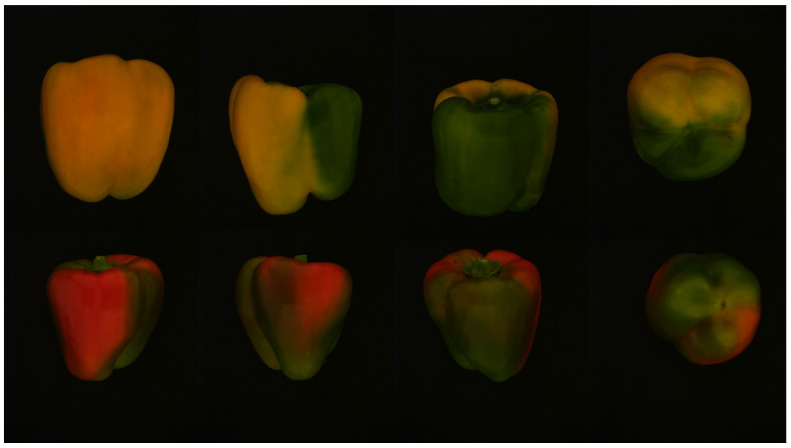
Example images of two peppers taken in the photocell.

Ninety-seven red sweet peppers (*cultivar: Banji; seed company: Efal*) and 102 yellow sweet peppers (*cultivar: Liri; Seed company: Hazera)* from maturity classes 1–4 were harvested from a commercial greenhouse in Kmehin, Israel, in January 2019. Each pepper was manually placed inside a photocell to ensure uniform illumination; each of the four photocell sides included three light-emitting diode (LED) spots of 35 watts each [47], resulting in total illumination of 49 lux [46]. Images were acquired using an IDS Ui-5250RE RGB color camera with a resolution of 1600 × 1200 pixels (IDS Imaging Development Systems GmbH, Obersulm, Germany), placed 38 cm above the black cell floor [46]. Images of each pepper were acquired from four viewpoints: three from the sides of the peppers, taken in no particular order, and the bottom viewpoint of the pepper (Figure 4).

The dataset, which is published as part of this paper, resulted in 796 RGB images of the different sides of the peppers and the whole pepper ground truth information of their maturity class (Table 1).

##### “Robotic” Dataset

Sixty-nine red and seventy yellow peppers from maturity classes 2, 3, and 4 (Table 1) were harvested from the same commercial greenhouse in Kmehin, Israel, in November 2019. No peppers were collected from class 1 since we assumed that immature peppers that are entirely green would not be detected by a harvesting robot [45,48,49].

**Figure 5:**
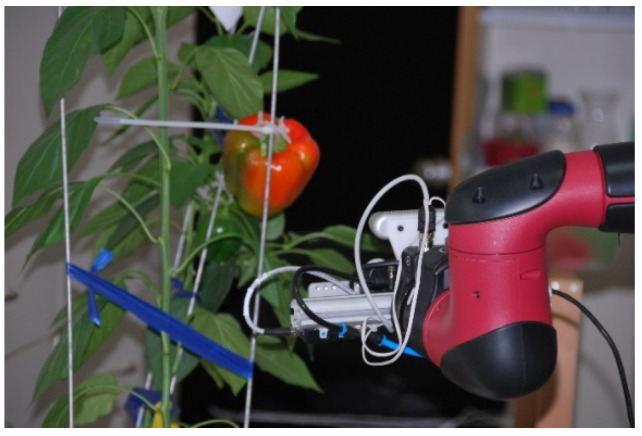
Camera mounted on the robot to acquire images of a red pepper fruit mounted on a pepper plant.

**Figure 6:**
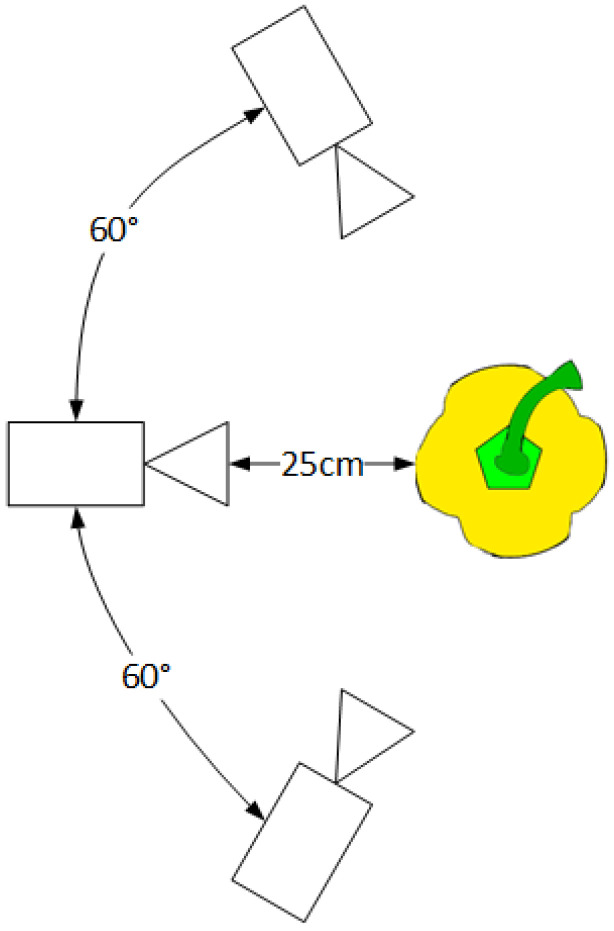
Top view of the viewpoints from which the images were acquired in the robotic experiments. All viewpoints have the same height.

Each pepper was individually attached to a pepper plant at a random orientation in a laboratory environment without controlled illumination (Figure 5). The peppers were attached to the plant hanging down straight in a way that does not create occlusion by leaves or stems and prevents overlap between peppers. Images for each pepper were acquired from three side viewpoints (Figure 6) from the same height. No images of the bottom viewpoint were taken since it is not always feasible for harvesting robots due to plant parts that prevent the robot from reaching the pepper from the bottom. The images were acquired using an Intel RealSense D435 RGB-D (Intel, Santa Clara, CA, USA) (color + depth) camera mounted on a Sawyer robotic 7-degree-of-freedom torque-controlled arm from Rethink Robotics [50]. Automatic exposure and white balance were enabled. The use of the robotic arm enables images from the three viewpoints to be made from the same pose for all peppers.

The dataset, which is published as part of this paper, resulted in 417 RGB-D images of the different sides of the peppers and the whole pepper ground truth information of their maturity class (Table 1).

##### “Orientation” Dataset

As part of the “robotic” dataset collection, 14 red peppers and 14 yellow peppers from maturity classes 2 and 3 (seven from each class) were taken from the peppers harvested for the robotic dataset. Each pepper was placed hanging straight down in a random orientation on a pepper plant in the laboratory. Images from three viewpoints were acquired for each pepper (Figure 3) three times, and each time the pepper was twisted 120° clockwise around the z-axis (regardless of the pepper shape), resulting in a different surface of the pepper facing the camera from the initial viewpoint. This collection produced a dataset of 252 RGB images.

### 2.2. Image Processing

Since the photocell dataset and the robotic/orientation datasets were taken in different illumination conditions, an individual segmentation process was created for each dataset. 

#### 2.2.1. “Photocell” Dataset Image Processing

The peppers from all the viewpoints were segmented and separated from the background using a previously developed blob detection algorithm [46]. The algorithm is based on an empirically selected threshold and the “Chan–Vese” active contour method [51], resulting in a binary image where each pixel was classified into a “pepper” class or “background” class (Appendix A). All image processing procedures were developed using MATLAB R2016a.

#### 2.2.2. Robotic and Orientation Datasets Image Processing

**Figure 7:**
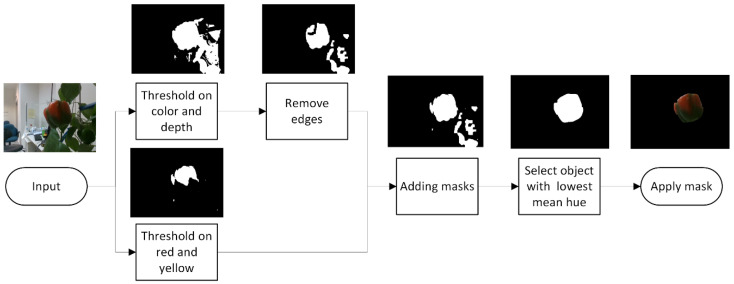
Segmentation of the pepper from the background.

A segmentation algorithm was developed to segment the peppers from the background in the laboratory environment with the following steps (Figure 7). Each RGB image was converted to an HSV—Hue Saturation Value color space, with the hue angle rotated 90° clockwise in order to create a continuous hue angle in the red-green color range [52]. Missing pixels in the depth image were filled using an onboard postprocessing step on the Intel RealSense camera by filling these pixels with the value of the closest neighboring pixel.

Segmenting the plants and peppers from the background was based on an empirically defined combination of the depth and color thresholds. All the regions that are in the defined color and depth range were retained, and the other parts were masked out (Appendix A). The resulting mask was refined using a morphological opening operation followed by a closing operation with a squared 3 × 3 structuring element.

Segmentation between pepper and plant was achieved using Canny edge detection on the value channel of the HSV image (Appendix A). To create a continuous shape, the edges around the detected pepper were dilated ten times using a 3 × 3 kernel, followed by five erosion operations. Since the Canny edge detection also removes the edges between the red/yellow color and the green color of the pepper, the red/yellow color is added back to the mask. The objects (connected components) in the mask are filtered based on the object size. From the remaining objects, the one with the lowest mean hue value is selected as the object belonging to the pepper. The convex hull of this object is used as the mask to segment the pepper from the background.

All image processing algorithms for segmentation were developed using OpenCV 3.3.1 in Python.

### 2.3. Feature Extraction and Maturity Level Classification 

#### 2.3.1. Feature Extraction

The following color features were extracted from three different color spaces: the hue dimension from the HSV color space, the red dimension from the RGB color space, and R–G, the difference between the red and green dimensions of the RGB color space [33,46,53,54,55]. The features from each color space included the following statistical features based on previous research [46], calculated on the pixel’s value of the segmented image: mean, standard deviation, minimal value, maximal value, median, and 5% trimmed mean (the mean calculated after discarding the lower and upper 5% of the data).

#### 2.3.2. Classification Algorithms

Five peppers for each class and each color were randomly selected for the test set (for each of the photocell and robotic experiments separately). The remaining peppers were used as the training set. An individual maturity level classification model was fit for each dataset and each pepper color (red/yellow) separately. The classification was implemented using a random forest algorithm that was found very useful in previous research [46]. Random forest is a robust and widespread algorithm that can be used for both regression and classification of multiple classes [56]. The algorithm is based on a large number of individual decision trees where each tree is fitted using different features chosen randomly and is independent of the other trees [56,57]. The implementation was based on 500 trees, with five features randomly chosen at each split. The orientation dataset images were classified using the classifier fitted for the robotic dataset since the images were acquired in the same condition and using the same peppers. The statistical analysis, model fitting, and classification algorithms were developed using R version 3.5.1 and RStudio 1.2.1135.

### 2.4. Analysis

In order to estimate the level of agreement between the viewpoint ground truth of the peppers, Cohen’s kappa coefficient was calculated [58]. This coefficient measures the inter-rater agreement for categorical data, defined as $\kappa =\frac{{\hat{P}}_{a}-{\hat{P}}_{e}}{1-{\hat{P}}_{e}}$, where ${\hat{P}}_{a}$ is the relative observed agreement among raters, and ${\hat{P}}_{e}\;$is the hypothetical probability of chance agreement, using the observed data to calculate the probabilities of each observer randomly classifying each class. This calculation will estimate the agreement between the information on a pepper from different viewpoints. The Light Kappa method for more than two raters was applied [59]. Since class 4 and class 1 peppers create an agreement between viewpoints by definition, since they are fully colored, they were extracted from the calculations, and the calculation was made only for peppers from classes 2 and 3.

The trained classification model was tested on the test set using information from different viewpoint combinations (Table 2). When more than one viewpoint was used for the classification, the pepper features were calculated by connecting all the pixels detected as peppers from those viewpoints into one vector before calculating the features. 

Classification accuracy (CA) was calculated to evaluate classification performance. It was defined as the number of peppers classified into the actual class of the whole pepper, divided by the total number of peppers.

Since, in a real-time application, the side viewpoint that will face the camera is unknown and cannot be predetermined, and since sweet peppers do not ripen in a uniform way, classification accuracy is highly dependent on the specific viewpoint and its relation to the overall maturity level of the pepper. Therefore, the range of accuracies that can be achieved on the test data using different specific viewpoints was estimated. The upper and lower bounds of this range were estimated using two scenarios with an example demonstrating their implementation (Table 3).

Optimistic scenario—the optimistic scenario assumes that the viewpoint that represents the pepper color level most accurately will face the robot camera. Classification performance in the optimistic scenario is calculated as follows: if one viewpoint (or one combination of viewpoints) classified the pepper into the right class, then it will be classified correctly in the optimistic scenario.

Pessimistic scenario—the pessimistic scenario assumes that, out of all potential viewpoints that could be used for classification, the viewpoint that represents the pepper color level the least accurately will face the robot camera. Therefore, in order to estimate the best classification performance in the pessimistic scenario, if one viewpoint (or one combination of viewpoints) classified the pepper into the wrong class, then it will be classified wrongly in the pessimistic scenario.

The goodness of fit of the classification algorithm concerning the current viewpoint information was analyzed by comparing the viewpoint classification to the ground truth for each viewpoint separately. This comparison was assessed using viewpoint classification accuracy (VCA), which is the number of viewpoints classified using the automatic classification into the same class as the viewpoint ground truth, divided by the total number of viewpoints.

## 3. Results and Discussion 

### 3.1. Analysis of “Photocell” Dataset

**Figure 8:**
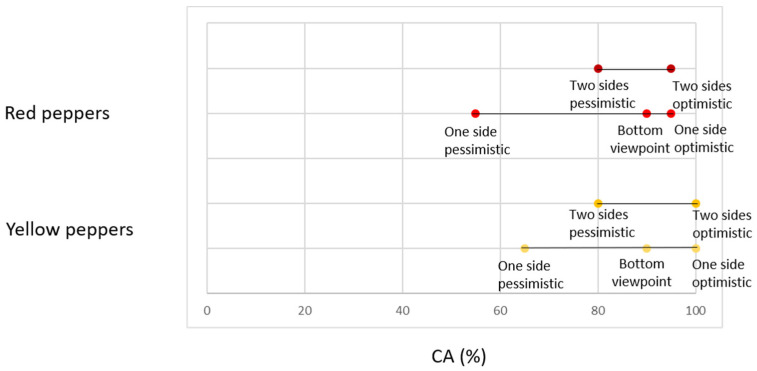
Classification accuracy for red and yellow peppers, “photocell” dataset.

For a single viewpoint, there is a big difference between the CA of the optimistic and pessimistic scenarios, with 35% and 40% differences for yellow and red peppers, respectively (Table 4, Figure 8, Appendix B). For two viewpoints, this difference is smaller, due to the higher pessimistic scenario accuracy. However, the two-viewpoint optimistic scenario resulted in the same CA as the single-viewpoint optimistic scenario, for both colors. These results show that two viewpoints ensure a higher chance of better classification, but still, in some cases, one viewpoint can yield the same or better results. However, this best viewpoint cannot be determined in advance.

Classification using the bottom viewpoint yielded results similar to the optimistic scenario (red *p*-value = 0.288, yellow *p*-value = 0.081) with only 5% and 10% difference between the classifications using the bottom viewpoint and the single-viewpoint optimistic scenario for red and yellow peppers, respectively (Table 4, Figure 8).

### 3.2. Analysis of “Robotic” Dataset

Analysis of the level of agreement between the viewpoints resulted in a value of 0.179 for Cohen’s kappa coefficient, indicating poor agreement for red peppers, and 0.258 for Cohen’s kappa coefficient, indicating fair agreement for yellow peppers [60]. Although this result might be irrelevant for a robotic harvester, it emphasizes the complexity of maturity level classification of sweet peppers and reveals the importance of viewpoint analysis. 

**Figure 9:**
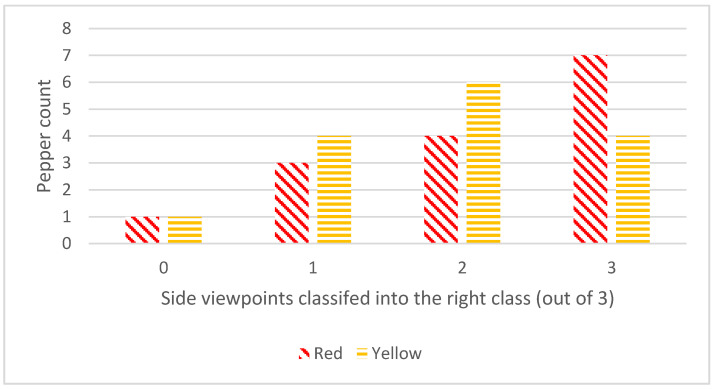
Frequency of the number of single viewpoints that were classified correctly.

Classification accuracy strongly depends on the pepper’s orientation on the plant, as seen in the range of results (Table 5, Figure 9, Appendix B). Misclassification was not a result of poor segmentation results (Appendix A).

**Figure 10:**
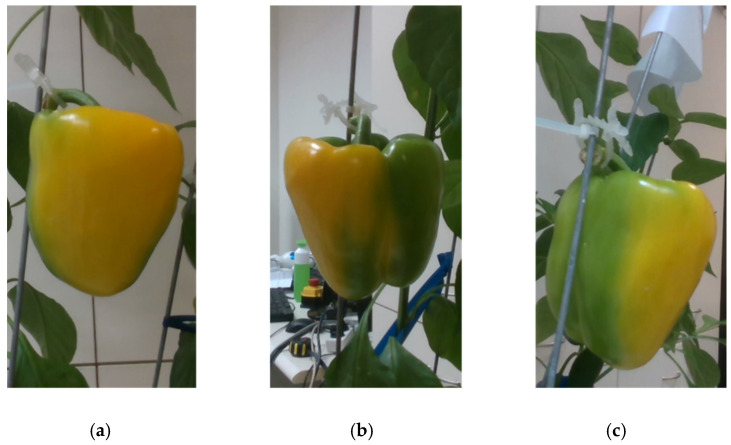
Example of incorrect classification after adding a viewpoint. A pepper from class 2 is classified correctly from the front viewpoint (**b**), but adding a viewpoint results in the wrong classification (into class 3) for both combinations of viewpoints, front + left (**a**,**b**) and front + right (**b**,**c**). When all three viewpoints are considered, classification is also correct.

Using two viewpoints resulted in better accuracy compared to a single viewpoint, as expected. However, the optimistic scenario for a single viewpoint outperforms classification when using a combination of viewpoints (for both, two or three viewpoints). This result can be explained by considering that, in some instances, the single viewpoint represents the color level of the pepper correctly, and by adding another viewpoint, the additional information may lead to the wrong classification, since it does not represent the full pepper. An example is demonstrated in Figure 10.

VCA of 71% was achieved for both red and yellow peppers. Two types of mismatch between the algorithm classification and the viewpoint’s ground truth occur. In the first type, the algorithm misclassifies both the whole pepper and the viewpoint ground truth. In the second type, the algorithm misclassifies the viewpoint’s ground truth but classifies the whole pepper correctly. For example, red pepper number 1 from class 2 was classified using the algorithm into class 2 from viewpoint 3; this is correct classification for the whole pepper, but the viewpoint ground truth classified this viewpoint into class 3, so this classification was counted as wrong for the VCA. This type of mismatch can be explained by the fact that the automated algorithm was trained based on the whole pepper classification and therefore takes into consideration information in the image that the human eye is not used to considering, like color intensity or the green level. In addition, this type of mismatch shows that, in some instances, the automated classification algorithm might yield better classification than manual sorting when using one viewpoint for the whole pepper.

### 3.3. Analysis of “Orientation” Dataset

Consistent classification among the three orientations was obtained for only 4 out of the 14 peppers, in both colors. For the other ten peppers, the classification algorithm yielded different classifications for the different pepper orientations. These results probably will be enhanced in field conditions where peppers are hung in different angles on the plant [61]. Future research might take the initial orientation of the pepper (or other fruit) on the plant into consideration; however, to derive this orientation, advanced image processing is needed. 

### 3.4. Comparison to Other Research 

Previous fruit maturity classification research resulted in similar classification accuracies (melon with 85.7% [62], banana with 87.1% [30], passion fruit with 91.5% [27], blueberry with 94% [36], papaya with 94.3% [40], date with 96.9% [39], and tomato with 99.31% [34]). However, this comparison is very limited due to several reasons; most of these fruits have uniform maturity patterns; all research were based on a single and usually random viewpoint of the fruit, and each research used a different classification method that varies in the amount of data needed and its computational complexity. Since each fruit has unique properties, comparing between results of different fruit might be irrelevant. 

Other more complex imaging technologies, such as hyperspectral imaging and nuclear and magnetic techniques, have been previously used for maturity classification of blueberry with 88–99% accuracy [63], cherry with 96.4% [38], and persimmon with 95.3% [64]. However, these advanced imaging technologies are still expensive and not applicable for robotic harvesting [3,65,66,67]. 

## 4. Conclusions

Classification is inconsistent between different views of the same pepper, indicating the importance of the viewpoint analyses for sweet pepper maturity classification. The bottom viewpoint is the best single viewpoint for maturity level classification. However, acquiring an image of the pepper from the bottom viewpoint is not always feasible, e.g., in the case of a harvesting robot due to plant parts that prevent the robot from reaching the pepper bottom viewpoint. From a single side viewpoint, classification accuracy is highly dependent on the fruit orientation, varying in the range of 46–95% for red peppers and 27–100% for yellow peppers. Hence, for sweet peppers, a single viewpoint is not sufficient for maturity classification. By using more than one viewpoint, the classification accuracy range can be significantly improved to 80–95% and 80–100% for red and yellow peppers, respectively. In the optimistic scenario, one viewpoint can yield better results than multiple viewpoints, but this is strongly dependent on the pepper’s orientation on the plant, which cannot be anticipated in advance. Therefore, ongoing work is aimed at developing an algorithm that dynamically decides if an additional viewpoint is needed, and if so, where this viewpoint should be. As maturity is a critical feature in selective harvesting, this will be important to improve. Future research can use the methods described in this paper for viewpoint analyses of other fruits. 

## Figures and Tables

**Table 1:** Yellow and red sweet pepper classes for the photocell and robotic experiments [46].

Class	Classification	Pepper Color	Photocell Dataset		Robotic Dataset	
			# Red Peppers	# Yellow Peppers	# Red Peppers	# Yellow Peppers
**1**	Immature	Green	22	21	-	-
**2**	Immature	Majority Green + Some Red/Yellow	26	26	23	23
**3**	Mature	Majority Red/Yellow + Some Green	24	29	24	26
**4**	Mature	Red/Yellow	25	26	22	21
**Total**			97	102	69	70

**Table 2:** Combinations of viewpoints tested.

Part	Using “Photocell” Dataset				Using “Robotic” Dataset		
# of viewpoints/ combination	1	2	3	4	1	2	3
	1 side	2 sides	3 sides	All four viewpoints	1 side	2 sides	3 sides
	Bottom	Bottom + 1 side	Bottom + 2 sides				

**Table 3:** Examples of optimistic and pessimistic scenarios results for correct and incorrect classifications using single viewpoints (VP). For two viewpoints, the same analysis was done but with the classification results of the viewpoints’ combination. CA is the classification accuracy.

Example #	VP1	VP2	VP3	Optimistic Scenario	Pessimistic Scenario
Pepper 1	Correct	Correct	Correct	Correct	Correct
Pepper 2	Correct	Incorrect	Correct	Correct	Incorrect
Pepper 3	Incorrect	Correct	Incorrect	Correct	Incorrect
Pepper 4	Incorrect	Incorrect	Incorrect	Incorrect	Incorrect
CA	50%	50%	50%	75%	25%

**Table 4:** CA achieved using a single viewpoint in comparison to all viewpoints for the “photocell” dataset.

Viewpoint Color	One-Viewpoint Pessimistic Scenario	One-Viewpoint Optimistic Scenario	Bottom Viewpoint	Two-Viewpoint Pessimistic Scenario	Two-Viewpoint Optimistic Scenario	All Viewpoints
Red pepper CA	55%	95%	90%	80%	95%	95%
Yellow pepper CA	65%	100%	90%	80%	100%	95%

**Table 5:** CA achieved for each of the viewpoint combinations for the robotic dataset.

Viewpoint Color	Viewpoint 1	Viewpoint 2	Viewpoint 3	One-Viewpoint Pessimistic Scenario	One-Viewpoint Optimistic Scenario	Combined Viewpoints 1 + 2	Combined Viewpoints 1 + 3	All Viewpoints
Red pepper CA	80%	60%	73%	46%	93%	80%	86%	86%
Yellow pepper CA	60%	60%	60%	27%	93%	67%	73%	80%

## Data Availability

The three datasets used for this paper were uploaded to Mendeley Data. Reserved DOI: 10.17632/ttntwwxkfd.1.

## Appendix A. Image Segmentation

### Appendix A.1. “Photocell” Dataset

Using the MATLAB image segmenter tool, the peppers were segmented and separated from the background using blob detection, with an empirically selected threshold of 10.

The “Chan–Vese” active contour method was applied for nine iterations on the segmented object [46,51].

Examples of the segmentation results can be seen in Table A1.

### Appendix A.2. Robotic and Orientation Dataset

#### Appendix A.2.1. Segmentation Parameters

The segmentation was done by using a combination of thresholds on depth and color with the following empirically selected thresholds for the segmentation in the rotated-HSV color space (Table A2).

**Table A1:** Segmentation results.

	Side Viewpoint		Bottom Viewpoint	
Paper Class	Image	Segmentation Mask	Image	Segmentation Mask
1				
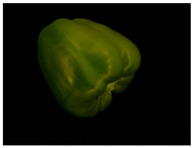
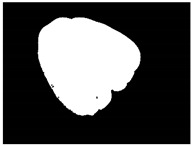
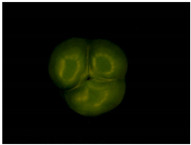
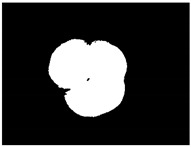
2				
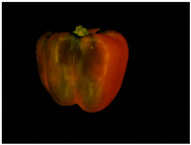
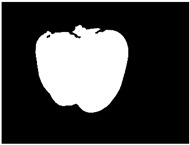
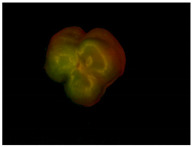
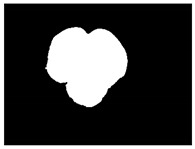
3				
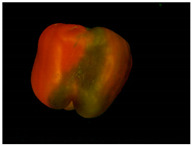
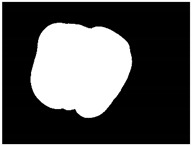
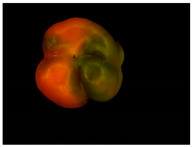
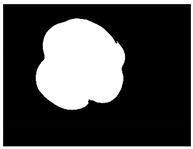
4				
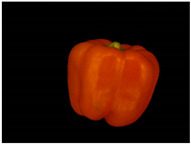
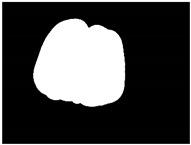
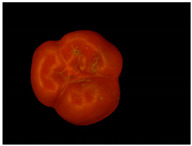
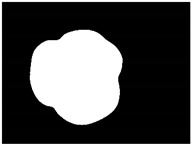
1				
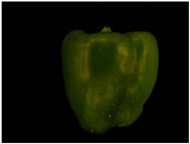
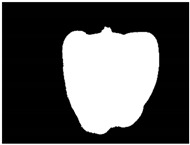
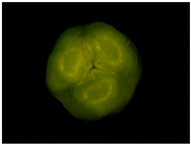
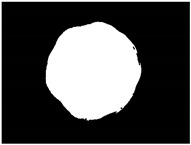
2				
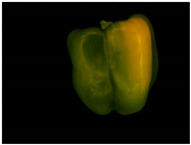
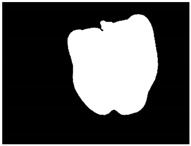
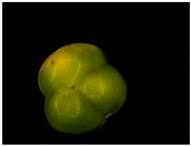
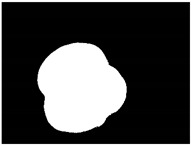
3				
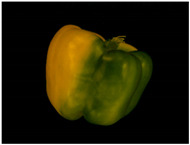
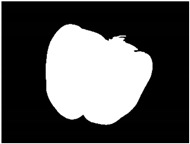
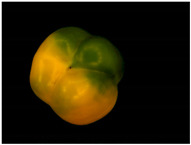
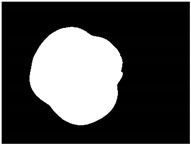
4				
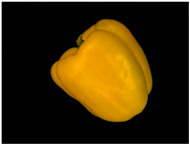
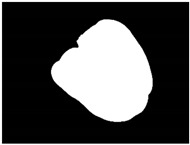
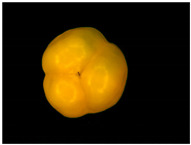
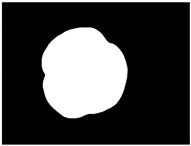

To make the Canny edge detection less dependent for the image brightness, the lower $t_{lower}$ and upper $t_{upper}$ detection thresholds are calculated by:

$$t_{lower}=\left(1-\sigma \right)*median\left(v\right)\;t_{upper}=\min\left(\left(1+\sigma \right)*median\left(v\right),\;255\right)$$

where σ=0.8 and v are the nonzero value channel values.

All objects with less than 5000 and more than 100,000 pixels are removed from the mask. The convex hull of the object with the highest mean hue is used as the mask for the pepper.

**Table A2:** Empirically selected thresholds for segmentation in rotated-HSV color space.

	Lower threshold			Upper Threshold		
Color	Hue	Saturation	Value	Hue	Saturation	Value
Green	83	62	0	150	255	255
Red	94	120	20	114	255	220
Yellow	109	155	0	115	255	255

### Appendix A.2.2. Segmentation results

The images below present examples of the peppers’ segmentation (Table A3). In the examples, at least one viewpoint is misclassified, except in the yellow pepper class 4 (because there was no misclassification). In each of the peppers, the segmentation was correct.

**Table A3:** Segmentation results.

	Front Viewpoint		Left Viewpoint		Right Viewpoint	
Pepper Class	Image	Segmentation Mask	Image	Segmentation Mask	Image	Segmentation Mask
2						
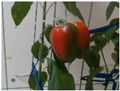
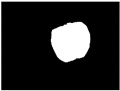
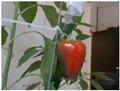
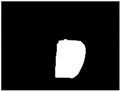
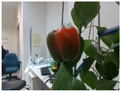
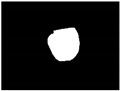
3						
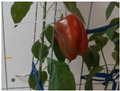
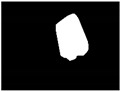
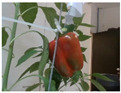
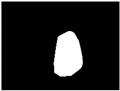
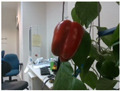
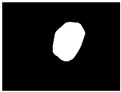
4						
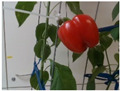
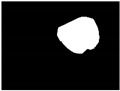
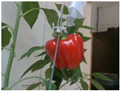
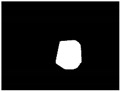
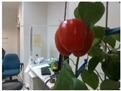
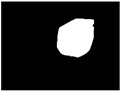
2						
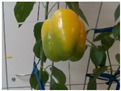
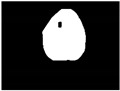
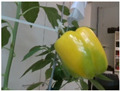
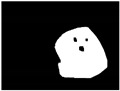
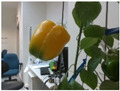
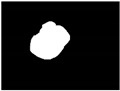
3						
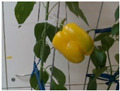
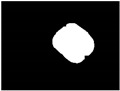
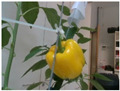
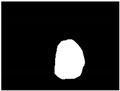
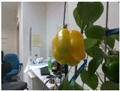
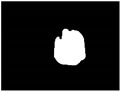
4						
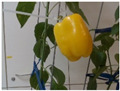
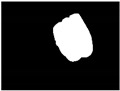
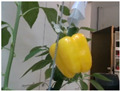
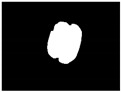
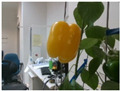
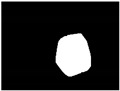

## Appendix B. Classification Confusion Matrixes

This appendix includes the confusion matrixes of the classification results for both *“photocell”* (Table A4, Table A5, Table A6, Table A7) and *“robotic”* datasets (Table A8, Table A9, Table A10, Table A11, Table A12, Table A13).

### Appendix B.1. “Photocell” Dataset

**Table A4:** Red peppers single viewpoints confusion matrixes for the *“Photocell” Dataset.*

Side Viewpoint ”Pessimistic”					Side Viewpoint “Optimistic”					Bottom Viewpoint				
	1	2	3	4		1	2	3	4		1	2	3	4
**1**	4	1	0	0	**1**	5	0	0	0	**1**	5	0	0	0
**2**	2	2	1	0	**2**	0	5	0	0	**2**	1	3	1	0
**3**	0	2	2	1	**3**	0	1	4	0	**3**	0	0	5	0
**4**	0	0	2	3	**4**	0	0	0	5	**4**	0	0	0	5
**CA**	55%				**CA**	95%				**CA**	90%			

**Table A5:** Red peppers multipole viewpoints confusion matrixes for the *“Photocell” Dataset.*

Two Viewpoints “Pessimistic”					Two Viewpoints “Optimistic”					All Viewpoints				
	1	2	3	4		1	2	3	4		1	2	3	4
**1**	5	0	0	0	**1**	5	0	0	0	**1**	5	0	0	0
**2**	0	4	1	0	**2**	0	5	0	0	**2**	0	4	1	0
**3**	0	1	2	2	**3**	0	1	4	0	**3**	0	0	5	0
**4**	0	0	0	5	**4**	0	0	0	5	**4**	0	0	0	5
**CA**	80%				**CA**	95%				**CA**	95%			

**Table A6:** Yellow peppers single viewpoints confusion matrixes for the *“Photocell” Dataset.*

Side Viewpoint ”Pessimistic”					Side Viewpoint “Optimistic”					Bottom Viewpoint				
	1	2	3	4		1	2	3	4		1	2	3	4
**1**	5	0	0	0	**1**	5	0	0	0	**1**	4	1	0	0
**2**	0	3	2	0	**2**	0	5	0	0	**2**	0	5	0	0
**3**	0	4	0	1	**3**	0	0	5	0	**3**	0	0	5	0
**4**	0	0	0	5	**4**	0	0	0	5	**4**	0	0	0	5
**CA**	65%				**CA**	100%				**CA**	95%			

**Table A7:** Red peppers multipole viewpoints confusion matrixes for the *“Photocell” Dataset.*

Two Viewpoints “Pessimistic”					Two Viewpoints “Optimistic”					All Viewpoints				
	1	2	3	4		1	2	3	4		1	2	3	4
**1**	5	0	0	0	**1**	5	0	0	0	**1**	5	0	0	0
**2**	0	4	1	0	**2**	0	5	0	0	**2**	0	4	1	0
**3**	0	3	2	0	**3**	0	0	5	0	**3**	0	0	5	0
**4**	0	0	0	5	**4**	0	0	0	5	**4**	0	0	0	5
**CA**	80%				**CA**	100%				**CA**	95%			

### Appendix B.2. “Robotic” Dataset

**Table A8:** Red peppers single viewpoints confusion matrixes for the *“Robotic” Dataset.*

Viewpoint 1				Viewpoint 2				Viewpoint 3			
	2	3	4		2	3	4		2	3	4
**2**	4	1	0	**2**	5	0	0	**2**	4	1	0
**3**	1	3	1	**3**	1	0	4	**3**	1	3	1
**4**	0	0	5	**4**	0	1	4	**4**	0	1	4
**CA**	90%			**CA**	60%			**CA**	73.3%		

**Table A9:** Red peppers single viewpoints scenario confusion matrixes for the *“Robotic” Dataset.*

One viewpoint “Pessimistic”				One Viewpoint “Optimistic”			
	2	3	4		2	3	4
**2**	3	2	0	**2**	4	1	0
**3**	1	0	4	**3**	1	4	0
**4**	0	1	4	**4**	0	0	5
**CA**	46.7%			**CA**	93.3%		

**Table A10:** Red peppers multipole viewpoints confusion matrixes for the *“Robotic” Dataset.*

Viewpoint 1 + 2				Viewpoint 1 + 3				All viewpoints			
	2	3	4		2	3	4		2	3	4
**2**	5	0	0	**2**	4	1	0	**2**	5	0	0
**3**	1	2	2	**3**	1	4	0	**3**	1	3	1
**4**	0	0	5	**4**	0	0	5	**4**	0	0	5
**CA**	80%			**CA**	86.7%			**CA**	86.7%		

**Table A11:** Yellow peppers single viewpoints confusion matrixes for the *“Robotic” Dataset.*

Viewpoint 1				Viewpoint 2				Viewpoint 3			
	2	3	4		2	3	4		2	3	4
**2**	1	3	1	**2**	3	0	2	**2**	3	2	0
**3**	0	3	2	**3**	2	1	2	**3**	1	4	1
**4**	0	0	5	**4**	0	0	5	**4**	0	2	4
**CA**	60%			**CA**	60%			**CA**	66.7%		

**Table A12:** Yellow peppers single viewpoints scenario confusion matrixes for the *“Robotic” Dataset.*

One viewpoint “Pessimistic”				One Viewpoint “Optimistic”			
	2	3	4		2	3	4
**2**	1	2	2	**2**	4	1	2
**3**	2	0	3	**3**	0	5	3
**4**	0	2	3	**4**	0	0	4
**CA**	26.7%			**CA**	93.3%		

**Table A13:** Yellow peppers multipole viewpoints confusion matrixes for the *“Robotic” Dataset.*

Viewpoint 1 + 2				Viewpoint 1 + 3				All viewpoints			
	2	3	4		2	3	4		2	3	4
**2**	2	2	1	**2**	3	2	0	**2**	2	3	0
**3**	1	3	1	**3**	1	4	0	**3**	0	5	0
**4**	0	0	5	**4**	0	1	4	**4**	0	0	5
**CA**	67.7%			**CA**	73.3%			**CA**	80%		
